# Qindan Capsule Attenuates Myocardial Hypertrophy and Fibrosis in Pressure Overload-Induced Mice Involving mTOR and TGF-*β*1/Smad Signaling Pathway Inhibition

**DOI:** 10.1155/2021/5577875

**Published:** 2021-04-28

**Authors:** Wenwu Bai, Min Ren, Wen Cheng, Xiaoting Lu, Deshan Liu, Bo Wang

**Affiliations:** ^1^Department of Traditional Chinese Medicine, Qilu Hospital, Cheeloo College of Medicine, Shandong University, Jinan, China; ^2^Key Laboratory of Cardiovascular Remodeling and Function Research of Ministry of Education, Qilu Hospital, Cheeloo College of Medicine, Shandong University, Jinan, China

## Abstract

Qindan capsule (QC), a traditional Chinese medicine compound, has been used to treat hypertension in the clinic for over 30 years. It is still not known about the effects of QC on pressure overload-induced cardiac remodeling. Hence, this study aims to investigate the effects of QC on pressure overload-induced cardiac hypertrophy, fibrosis, and heart failure in mice and to determine the possible mechanisms. Transverse aortic constriction (TAC) surgery was used to induce cardiac hypertrophy and heart failure in C57BL/6 mice. Mice were treated with QC or losartan for 8 weeks after TAC surgery. Cardiac function indexes were evaluated with transthoracic echocardiography. Cardiac pathology was detected using HE and Masson's trichrome staining. Cardiomyocyte ultrastructure was detected using transmission electron microscopy. Hypertrophy-related fetal gene expression was investigated using real-time RT-PCR. The expression of 8-OHdG and the concentration of MDA and Ang-II were assessed by immunohistochemistry stain and ELISA assay, respectively. The total and phosphorylated protein levels of mTOR, p70S6K, 4EBP1, Smad2, and Smad3 and the expression of TGF-*β*1 and collagen I were measured using western blot. The results showed that low- and high-dose QC improved pressure overload-induced cardiac hypertrophy, fibrosis, and dysfunction. QC inhibited ANP, BNP, and *β*-MHC mRNA expression in failing hearts. QC improved myocardial ultrastructure after TAC surgery. Furthermore, QC downregulated the expression of 8-OHdG and the concentration of MDA, 15-F_2t_-IsoP, and Ang-II in heart tissues after TAC surgery. We also found that QC inhibited the phosphorylation of mTOR, p70S6K, and 4EBP1 and the expression of TGF-*β*1, p-Smad2, p-Smad3, and collagen I in pressure overload-induced failing hearts. These data indicate that QC has direct benefic effects on pressure overload-induced cardiac hypertrophy, fibrosis, and dysfunction. The protective effects of QC involve prevention of increased oxidative stress injury and Ang-II levels and inhibition of mTOR and TGF-*β*1/Smad pathways in failing hearts.

## 1. Introduction

Hypertension- or aortic stenosis-induced left ventricular pressure overload leads to heart failure or sudden death in clinical practice [1]. Myocardial hypertrophy induced by pressure overload develops from compensation to decompensation. These developments of myocardial remodeling are characterized by hypertrophic cardiomyocytes, upregulated expression of fetal genes, activated protein synthesis, extracellular matrix deposition, and fibrosis [2]. Although the cardiac remodeling represents that the heart tries to maintain the contractile function, it is getting worse under sustained pressure overload stress and eventually leads to heart failure. Nevertheless, the underlying mechanism mediating the pathological process of cardiac hypertrophy and fibrosis is complex and involves multifarious regulators and signaling pathways.

Clinically, angiotensin-converting enzyme inhibitors, *β* receptor blockers, diuretics, angiotensin II (Ang II) receptor blockers, and angiotensin receptor-neprilysin inhibitors are used to relieve symptoms and reduce the mortality of patients with heart failure [3]. On the basis of traditional Chinese medicine theory, heart failure belongs to a category of Qi deficiency, and it has been treated with herbs for thousands of years in China. Recently, some clinical and experimental studies have shown that Chinese herbal medicine has the beneficial effects on heart failure [4, 5]. Nevertheless, the specific mechanism remains unclear.

Qindan capsule (QC) is a traditional Chinese medicine compound. As previously reported, it is composed of *Scutellaria baicalensis* Georgi, *Salvia miltiorrhiza* Bge, *Coptis chinensis* Franch, *Uncaria rhynchophylla* (Miq.) Miq. ex Havil, *Ligusticum striatum* DC, *Leonurus japonicus* Houtt, and *Scurrula parasitica* L. [6]. QC is used to treat hypertension in the clinic, and clinical studies showed that QC improves the prothrombotic state and balances the levels of blood endothelin, calcitonin gene-related peptide, and Ang II in patients with primary hypertension induced by Yang hyperactivity and blood stagnation [7, 8]. We have demonstrated that in spontaneous hypertensive rats (SHR), QC ameliorates vascular remodeling through inhibition of basic fibroblast growth factor and osteopontin expression [6, 9, 10]. We also found that QC could inhibit the TGF-*β*1/Smad3 pathway in vascular adventitial fibroblasts and thus reduce cell proliferation and collagen synthesis [11–13]. However, whether QC can be used to alleviate pressure overload-induced myocardial hypertrophy, fibrosis, and heart failure is still unknown. Here, we evaluated the effects of QC in different doses on myocardial remodeling and function in the transverse aortic constriction (TAC) surgery-induced heart failure mice model and attempted to investigate the underlying mechanism.

## 2. Materials and Methods

### 2.1. Preparation and Quality Evaluation of QC

The formulation of QC is listed in Table S1, which was authenticated and standardized to marker compounds according to the Chinese Pharmacopoeia 2020 [14]. We prepared QC as previously described [11]. Briefly, raw herbs of QC in proportion were soaked in water first and then decocted with 12 times (volume/weight) distilled water for three times. The supernatant was filtrated and dried to powder using a vacuum drier. The dried powder was 25% (weight/weight) according to the original materials, which was diluted with distilled water to the required concentration prior to use.

Previously, we established the quality evaluation of QC using high-performance liquid chromatography (HPLC; LC-2010HT, Shimadzu, Japan) [11]. Five major active constituents were identified from QC, including baicalin (19.11 mg/g), 3,4-dihydroxyphenyllactic acid (0.669 mg/g), berberine (4.3 mg/g), rhynchophylline (0.126 mg/g), and stachydrine (30.1 mg/g) (Figure S1).

### 2.2. Animals and TAC Surgery

Twelve-week-old male C57BL/6 mice were bought from Vital River Laboratory Animal Technologies Co. Ltd (Beijing, China). TAC surgery was performed as previously described [15]. Anesthesia was performed using ketamine (20 mg/kg) and xylazine (1 mg/kg). Then, a ventilator was used to artificially maintain anesthetized mice respiration. The transverse aorta was accessed via a sternotomy, and constriction of the left common carotid artery was performed using a 27-G needle. Mice in the sham operation group (*n* = 12) underwent the similar surgical procedures without aortic constriction.

Three days after TAC surgery, mice were randomly divided into four groups (*n* = 12 for each group): TAC group, TAC with low-dose QC group (TAC + QCL, mice were fed orally with 0.3 g/kg per day QC powder), TAC with high-dose QC group (TAC + QCH, mice were fed orally with 1.5 g/kg per day QC powder), and TAC with losartan group (TAC + LST (MSD & Co, Inc., Hangzhou, China), mice were fed orally with 0.03 g/kg per day losartan). Equal volumes of distilled water were used for mice in the sham and TAC groups. Eight weeks after surgery, hearts of the above-mentioned mice were harvested for weighing and further assays. All animal experiments were performed at the Key Laboratory of Cardiovascular Remodeling and Function Research and approved by the local ethics committee (DWLL-2017-017).

### 2.3. Echocardiography

Transthoracic echocardiography was conducted using a Vevo 770 imaging system equipped with a 30-MHz transducer (VisualSonics, Canada). The mice anesthesia was performed as described previously [15]. Under M-mode echocardiography, the left ventricular posterior wall at diastole (LVPWd), left ventricular internal dimension at diastole (LVIDd), and left ventricular internal dimension at systole (LVIDs) were measured. Then, percentage fractional shortening (FS%) and percentage ejection fraction (EF%) were calculated using a formula as previously described [16]. Transthoracic echocardiography was performed right before surgery (served as baseline) and 4 weeks and 8 weeks after surgery.

### 2.4. Histology and Immunohistochemistry (IHC) Assay

The freshly isolated hearts were fixed in paraformaldehyde. Then, tissue sections were obtained and stained using hematoxylin and eosin (HE) to assess histopathology or using Masson's trichrome with standard procedures to evaluate cardiac fibrosis. Antibody against 8-hydroxydeoxyguanosine (8-OHdG; Abcam, UK) was used for IHC stain. Mean cross-sectional area of the myocytes was measured in HE-stained transverse sections, and cardiac fibrosis degree was analyzed in Masson's trichrome-stained sections using Image-Pro Plus 6.0 (Media Cybernetics, USA). The mean 8-OHdG-positive cells were calculated under ×400 magnification. For each analysis, 10 random fields were selected under the microscope for each sample.

### 2.5. Transmission Electron Microscopy (TEM)

Detection of cardiac ultrastructure was performed as described previously [16]. Briefly, freshly isolated heart tissues in the size of 0.5 mm × 1 mm × 5 mm were fixed with glutaraldehyde (2%) overnight. After washed by 0.2 M PBS for 3 times, tissue pieces were fixed with 1% osmium tetraoxide, washed, and dehydrated using gradient concentrations of ethanol. Then, the fixed heart tissues were soaked in Epon812 resin/acetone (1 : 1), heated to 70°C, and embedded overnight. Slices with thickness of 50 nm were made using an ultramicrotome LKB-8800 (LKB-Produkter AB, Bromma, Sweden). Ultrastructure pathological changes in cardiomyocytes including mitochondria and sarcomeres were recorded using TEM H-7000FA (Hitachi, Japan). The mitochondrial volume density (MitoVD) was quantified using Image-Pro Plus 6.0.

### 2.6. ELISA

Heart tissues were harvested, homogenated, and then examined using ELISA kits (Elabscience, Wuhan, China) to detect malondialdehyde (MDA) and Ang-II concentrations. 15-F_2t_-isoprostane (15-F_2t_-IsoP) in heart tissue homogenates was measured using ELISA kits (Cayman Chemical, MI, USA). The ELISA assays were performed according to the manufacturer's instructions.

### 2.7. Real-Time PCR

Heart tissues were harvested. Total RNA was isolated with TRIzol (Invitrogen, USA) and then converted into cDNA using PrimeScript^™^ 1^st^ Strand cDNA Synthesis Kit (Takara Biotechnology, Japan). Real-time PCRs were conducted for the following genes—atrial natriuretic peptide (ANP), *β*-myosin heavy chain (*β*-MHC), and brain natriuretic peptide (BNP)—using a thermocycler (iQ5; Bio-Rad, USA). The primer sequences used in this study are listed in Table S2. The expression levels of the above genes were normalized to GAPDH using the 2^−ΔΔCT^ method.

### 2.8. Western Blot

Fresh heart tissues from mice were harvested and lysed using RIPA lysing buffer (Beyotime, Shanghai, China) to extract total protein. The primary antibodies were listed as follows: anti-GAPDH, anti-mTOR, anti-phosphorylated-mTOR (Ser2448) (p-mTOR), anti-p70S6K, anti-p-p70S6K (Thr389), anti-4EBP1, anti-p-4EBP1(Ser65), anti-Smad2, anti-p-Smad2 (Ser465/467), anti-Smad3, and anti-p-Smad3 (Ser423/425) (Cell Signaling Technology, USA); anti-TGF-*β*1 (Santa Cruz Biotechnology, USA); and anti-collagen I (Abcam, UK). Signals were detected using the FluorChem E data system (Cell Biosciences, USA) and then quantified using Quantity One 4.52 (Bio-Rad, USA).

### 2.9. Statistic Analysis

All statistical data of this study were presented as mean ± SEM, and the statistical analyses were performed using GraphPad Prism 8.0 software (GraphPad Software Inc., USA). One-way ANOVA was performed to compare the differences among multiple groups. *P* values less than 0.05 were considered statistically significant.

## 3. Results

### 3.1. QC Improved Cardiac Function after TAC Surgery

**Figure 1:**
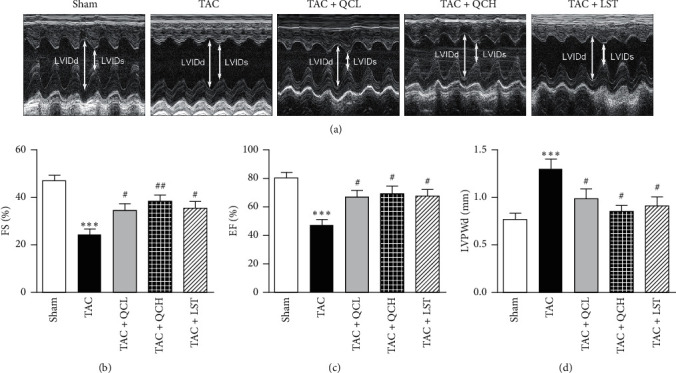
Effects of QC on cardiac function after TAC surgery in mice. (a) Transthoracic echocardiography of mice in the indicated groups 8 weeks after TAC surgery. (b) Fractional shortening (FS%), (c) ejection fraction (EF%), and (d) LV posterior wall at diastole (LVPWd) were evaluated. ^*∗∗∗*^*P* < 0.001 vs. sham group. ^#^*P* < 0.05 and ^##^*P* < 0.01 vs. TAC group. Data are presented as mean ± SEM and *n* = 12.

We used transthoracic echocardiography to evaluate the cardiac systolic function at different time points. Four weeks after TAC surgery, increased LVPWd was alleviated in the TAC + QCH group compared with the TAC group (Table S3). Then, 8 weeks after TAC surgery, decreased FS%, EF%, and increased LVPWd were significantly rescued in TAC + QCL, TAC + QCH, and TAC + LST groups compared with the TAC group (Table S3, Figures 1(a)–1(d)). No significant difference was observed between QC- and LST-treated groups (Table S3, Figures 1(a)–1(d)). Our data suggest that QC, no matter the low or high dose, has the protective effects on pressure overload-induced cardiac dysfunction in mice.

### 3.2. QC Ameliorated Myocardial Hypertrophy and Fibrosis after TAC Surgery

**Figure 2:**
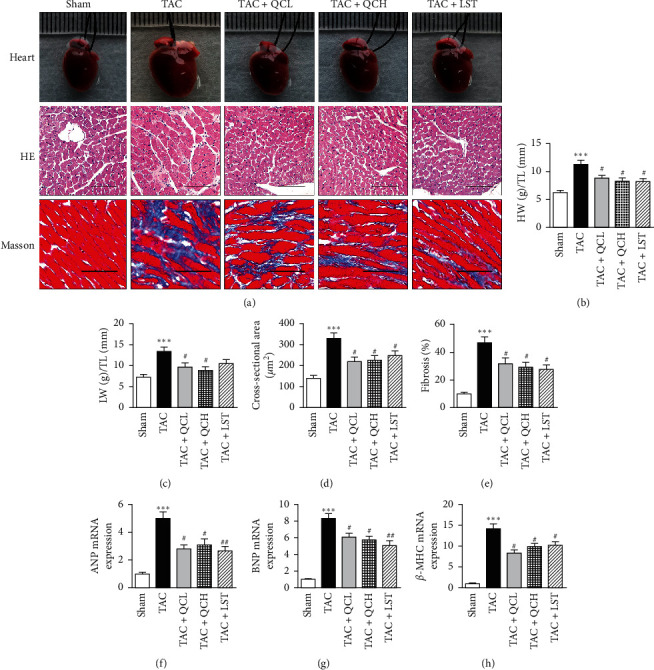
Effects of QC on myocardial hypertrophy and fibrosis after TAC surgery in mice. (a) Representative images of hearts, HE stain, and Masson's trichrome staining of mice left ventricles at the end of 8 weeks after TAC surgery. Scale bar, 50 *μ*m. (b) Ratios of heart weight to tibial length (HW/TL) and (c) ratios of lung weight to tibial length (LW/TL) were measured 8 weeks after TAC surgery. (d) Cross-sectional area of cardiomyocytes was quantified with HE sections. (e) Cardiac fibrosis area was quantified with Masson's trichrome-stained sections. Relative mRNA levels of (f) ANP, (g) BNP, and (h) *β*-MHC were evaluated using RT-PCR. ^*∗∗∗*^*P* < 0.001 vs. sham group. ^#^*P* < 0.05 and ^##^*P* < 0.01 vs. TAC group. Data are presented as mean ± SEM and *n* = 5.

Here, the analysis of morphology and weight of heart tissue was used to show the degree of myocardial hypertrophy and fibrosis after TAC surgery. At 8 weeks postsurgery, QC and LST prevented cardiac dilatation induced by TAC surgery (Figure 2(a)). The ratios of heart weight to tibial length and lung weight to tibial length were significantly lower in TAC + QCL and TAC + QCH groups compared with the TAC group (Figures 2(b) and 2(c)). The treatment of QC and LST significantly decreased the cardiomyocyte cross-sectional area after TAC surgery (Figure 2(d)). The collagen volume fraction was evaluated using Masson's trichrome staining to analyze the cardiac fibrosis degrees after TAC. At 8 weeks postsurgery, when compared with the TAC group, the collagen volume fraction decreased significantly in TAC + QCL, TAC + QCH, and TAC + LST groups (Figure 2(e)). Myocardial mRNA levels of ANP, *β*-MHC, and BNP, acting as markers of myocardial hypertrophy, were investigated in this study. The mRNA levels of ANP, *β*-MHC, and BNP increased after TAC surgery and were significantly reversed in TAC + QCL, TAC + QCH, and TAC + LST groups (Figures 2(f)–2(h)). Our results indicate that treatment with QC attenuated pressure overload-induced myocardial hypertrophy and fibrosis in mice.

### 3.3. QC Improved Myocardial Ultrastructure after TAC Surgery

**Figure 3:**
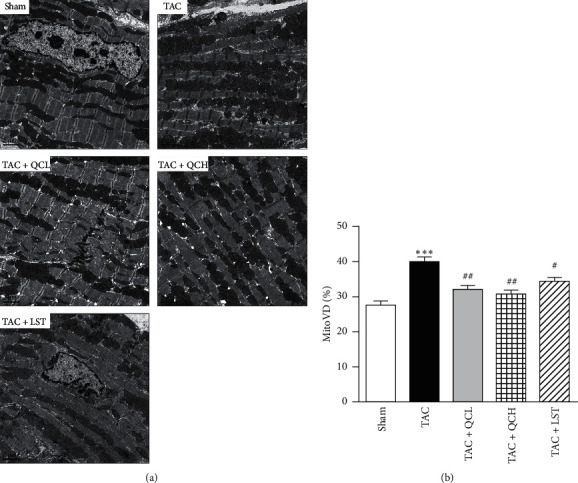
Effects of QC on myocardial ultrastructure after TAC surgery in mice. (a) TEM of cardiomyocytes from the indicated groups. Scale bar, 2 *μ*m. (b) Quantification of mitochondrial volume density (MitoVD). ^*∗∗∗*^*P* < 0.001 vs. sham group. ^#^*P* < 0.05 and ^##^*P* < 0.01 vs. TAC group. Data are presented as mean ± SEM and *n* = 3.

Myocardial ultrastructure was analyzed using TEM, and obvious structural changes were observed in failing hearts postsurgery including severe swelling and extensive vacuolization in the mitochondria (Figure 3(a)). Stereological quantification revealed a significant decrease of MitoVD in TAC + QCL, TAC + QCH, and TAC + LST groups compared with the TAC group (Figure 3(b)). These data suggest that treatment with QC ameliorated the changes of myocardial ultrastructure induced by TAC surgery.

### 3.4. QC Attenuated Cardiac Oxidative Stress Injury and Ang-II Content after TAC Surgery

**Figure 4:**
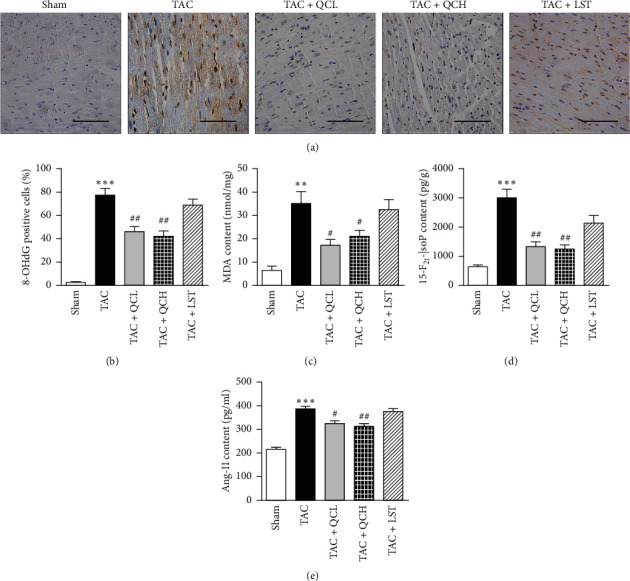
Effects of QC on cardiac oxidative stress injury and Ang-II content after TAC surgery in mice. (a) IHC-stained sections of 8-OHdG; scale bar, 50 *μ*m. (b) Quantification of 8-OHdG-positive cells in indicated groups. (c) Levels of malondialdehyde (MDA), (d) 15-F_2t_-IsoP, and (e) Ang-II were quantified in heart tissues of mice. ^*∗∗*^*P* < 0.01 and ^*∗∗∗*^*P* < 0.001 vs. sham group; ^#^*P* < 0.05 and ^##^*P* < 0.01 vs. TAC group. Data are presented as mean ± SEM and *n* = 5.

As biomarkers of oxidative stress injury in the heart, levels of 8-OHdG, MDA, and 15-F_2t_-IsoP were analyzed using HIC and ELISA assay, respectively. Expression of 8-OHdG and concentrations of MDA and 15-F_2t_-IsoP decreased significantly in TAC + QCL and TAC + QCH groups, but not in the TAC + LST group, compared with the TAC group (Figures 4(a)–4(d)). Then, we detected Ang-II concentration in heart tissue homogenates using ELISA assay in this study. Compared with the TAC group, Ang-II concentration decreased significantly in TAC + QCL and TAC + QCH groups, but not in the TAC + LST group (Figure 4(e)). These data suggest that treatment of QC, but not LST, attenuated cardiac oxidative stress injury and Ang-II content after TAC surgery.

### 3.5. QC Inhibited mTOR and TGF-*β*1/Smad Pathways in the Mice Heart after TAC Surgery

**Figure 5:**
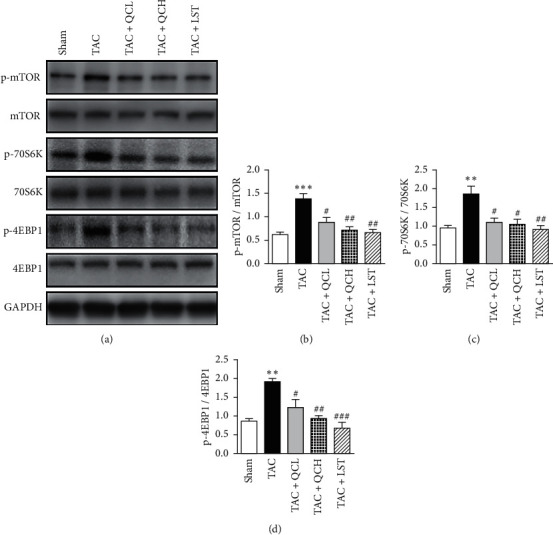
Effects of QC on the cardiac mTOR signaling pathway after TAC surgery in mice. (a) Representative results of protein levels and phosphorylation levels of mTOR, 70S6K, and 4EBP1. Quantification of ratios of p-mTOR to mTOR (b), p-70S6K to 70S6K (c), and p-4EBP1 to 4EBP1 (d). ^*∗∗*^*P* < 0.01 and ^*∗∗∗*^*P* < 0.001 vs. sham group. ^#^*P* < 0.05, ^##^*P* < 0.01, and ^###^*P* < 0.001 vs. TAC group. Data are presented as mean ± SEM and *n* = 3.

**Figure 6:**
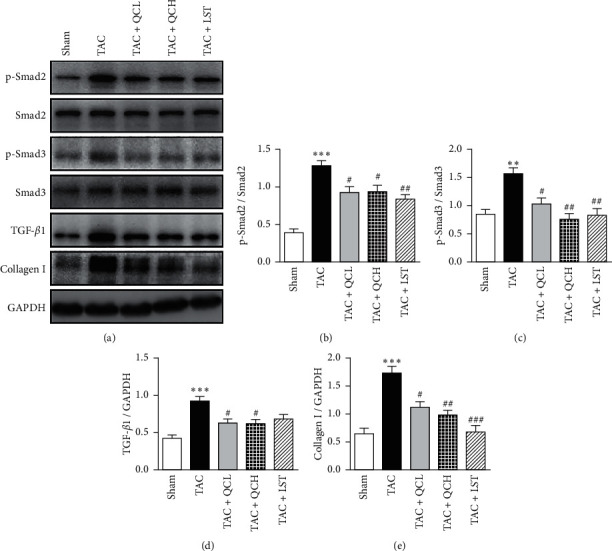
Effects of QC on the cardiac TGF-*β*1/Smad pathway after TAC surgery in mice. (a) Representative results of the protein levels of TGF-*β*1, collagen I, and GAPDH and phosphorylation levels of Smad2 and Smad3. Quantification of ratios of p-Smad2 to Smad2 (b), p-Smad3 to Smad3 (c), TGF-*β*1 to GAPDH (d), and collagen I to GAPDH (e). ^*∗∗*^*P* < 0.01 and ^*∗∗∗*^*P* < 0.001 vs. sham group. ^#^*P* < 0.05, ^##^*P* < 0.01, and ^###^*P* < 0.001 vs. TAC group. Data are presented as mean ± SEM and *n* = 3.

To explore the underlying mechanism of QC-mediated protection of cardiac function after TAC surgery, we first investigated the protein levels and phosphorylation levels of mTOR and its targets S6 kinase (S6K) and eIF4E-binding protein-1 (4EBP1) using western blot. At 8 weeks postsurgery, the phosphorylation levels of mTOR, 70S6K, and 4EBP1 significantly decreased in TAC + QCL, TAC + QCH, and TAC + LST groups compared with the TAC group (Figure 5). Next, we investigated the protein levels of TGF-*β*1 and its downstream Smad. At 8 weeks postsurgery, the protein levels of TGF-*β*1 and collagen I and phosphorylation levels of Smad2 and Smad3 were upregulated in the TAC group compared with the sham surgery group (Figure 6). The treatment using low- and high-dose QC, as well as LST, inhibited the protein levels of TGF-*β*1 and collagen I and phosphorylation levels of Smad2 and Smad3 significantly (Figure 6). These data suggest that QC inhibited the increased activities of mTOR and TGF-*β*1/Smad signaling pathways in pressure overload-induced failing hearts.

## 4. Discussion

In this study, our findings illustrated that low- and high-dose QC improved pressure overload-induced myocardial hypertrophy, fibrosis, and heart failure. QC inhibited mRNA levels of ANP, *β*-MHC, and BNP in failing hearts. QC improved myocardial ultrastructure after TAC surgery. Furthermore, QC downregulated the expression of 8-OHdG and the content of MDA, 15-F_2t_-IsoP, and Ang-II in heart tissues after TAC surgery. We also found that QC inhibited the protein levels of TGF-*β*1 and collagen I and phosphorylation levels of mTOR, p70S6K, 4EBP1, Smad2, and Smad3 in pressure overload-induced failing hearts.

mTOR, which belongs to the phosphoinositide kinase-related kinase family, functions as an atypical serine/threonine kinase. It has been proven that mTOR plays an important role in myocardial hypertrophy [17]. Here, our results showed that not only the phosphorylation of mTOR but also its downstream proteins p70S6K and 4EBP1 increased in pressure overload-induced heart failure. This is in consistence with previous studies [18]. It suggests that mTOR is activated in response to pressure overload stress. Activated mTOR phosphorylates its substrates S6K and 4EBP1 to induce protein synthesis and hypertrophy in cardiomyocytes [19]. Inhibition of mTOR activation is beneficial in pressure overload-induced myocardial hypertrophy [20]. Here, our results demonstrated that QC inhibited phosphorylation of mTOR, p70S6K, and 4EBP1 in the heart. This indicates that the protective effects of QC on myocardial hypertrophy involve the inhibition of mTOR signaling pathway.

We previously reported that QC inhibits proliferation of aortal adventitial fibroblasts and decreases collagen synthesis through inhibiting the TGF-*β*1/Smad3 pathway [11]. TGF-*β*1 has been verified to act as a major regulator in promoting cardiovascular remodeling induced by hemodynamic overload [21]. The upregulated expression of TGF-*β*1 correlates with the levels of cardiac fibrosis in pressure overload-induced hypertrophy [22]. Our data also showed that increased levels of TGF-*β*1 in failing hearts after TAC surgery. TGF-*β*1 induces extracellular matrix production including collagen and fibronectin, stimulates myofibroblasts migration and proliferation, and promotes fetal gene expression in hypertrophic hearts through Smad proteins phosphorylation [23]. The inhibition of TGF-*β*1/Smad pathway has been shown to account for the attenuation of cardiac hypertrophy both *in vivo* and *in vitro* [24]. In this study, we found that QC treatment decreased the protein levels of TGF-*β*1 and the phosphorylation of Smad2/3 in hypertrophic hearts. Thus, the QC-mediated inhibition of mTOR and TGF-*β*1/Smad pathways may account for improvement of heart function in the pressure overload-induced model of heart failure.

It has been reported that reactive oxygen species (ROS) promote mTOR phosphorylation through activating AMPK [25] and activates the TGF-*β*1/Smad3 pathway through HIF-1*α* [26]. Antioxidants attenuate pathological cardiac hypertrophy through inhibiting mTOR [27] or TGF-*β*1 [28]. We found that QC attenuated oxidative stress injury in failing hearts. Actually, baicalin, one of the active constituents in QC, has been proved to exert antioxidant effect [29]. It suggests that QC inhibits mTOR and TGF-*β*1/Smad pathways through its antioxidant effect.

It is also known that the renin-angiotensin system (RAS) plays an important role in cardiac remodeling and the beneficial effects of its inhibition on cardiac hypertrophy and dysfunction. Both mTOR and TGF-*β*1/Smad pathways have been shown to be activated by Ang-II in heart failure [30, 31]. Our previous study demonstrated that QC ameliorates the increased levels of Ang-II in plasma and vascular tissues in SHR [10]. Here, our data demonstrated that QC decreased the levels of Ang-II in hearts after TAC, which might contribute to the inhibition of mTOR and TGF-*β*1/Smad pathways.

It is well known that compound preparations of traditional Chinese medicine contain a large number of chemical substances and active ingredients. According to the main active ingredients of QC detected by HPLC, baicalin inhibits pressure overload-induced cardiac fibrosis through regulating the AMPK/TGF-*β*/Smads signaling pathway [32]. Berberine alleviates pressure overload-induced cardiac hypertrophy and dysfunction through the inhibition of mTOR and p38 MAPK signaling pathways associated with enhanced autophagy [33]. 3,4-dihydroxyphenyllactic acid protects against cardiac dysfunction and fibrosis following myocardial infarction through decreasing angiotensin-converting enzyme expression and Ang II content [34]. Stachydrine relieves pressure overload-induced cardiac hypertrophy by reducing the generation of ROS [35] and inhibiting the Ang II/TGF-*β*1 axis [36]. Therefore, these reports indicate that the observed improvement of QC on pressure overload-induced failing hearts and regulation of related molecular signaling pathways could be a synergistic effect of multiple active ingredients.

## 5. Conclusion

Our results indicate that QC exerts direct beneficial effects on pressure overload-induced myocardial hypertrophy, fibrosis, and dysfunction. The beneficial effects of QC include prevention of increased oxidative stress injury and Ang-II content and inhibition of the mTOR signaling and the TGF-*β*1/Smad pathway in failing hearts.

## Data Availability

The data used to support the findings of this study are available from the corresponding author upon request.

## Abbreviations

4EBP1: eIF4E-binding protein-1
8-OhdG: 8-Hydroxydeoxyguanosine
15-F_2t_-isoprostane: 15-F_2t_-IsoP
ANP: Atrial natriuretic peptide
BNP: Brain natriuretic peptide
*β*-MHC: *β*-Myosin heavy chain
EF: Ejection fraction
FS: Fractional shortening
GAPDH: Glyceraldehyde-3-phosphate dehydrogenase
HE: Hematoxylin and eosin
HPLC: High-performance liquid chromatography
LVIDd: Left ventricular internal dimension at diastole
LVIDs: Left ventricular internal dimension at systole
LVPWd: Left ventricular posterior wall at diastole
MDA: Malondialdehyde
MitoVD: Mitochondrial volume density
QC: Qindan capsule
RAS: Renin-angiotensin system
ROS: Reactive oxygen species
S6K: S6 kinase
SHR: Spontaneous hypertensive rats
TAC: Transverse aortic constriction
TEM: Transmission electron microscopy.

## Supplementary Materials

Supplementary Materials Figure S1: quality evaluation of QC using HPLC: (a) baicalin; (b) 3,4-dihydroxyphenyllactic acid; (c) berberine; (d) rhynchophylline; and (e) stachydrine. Table S1: recipe of Qindan capsule (QC) formulation. Table S2: mouse primers used for real-time RT-PCR. Table S3: echocardiographic parameters in different time points. Cardiac functional parameters measured by transthoracic echocardiography at week 0 (baseline), week 4, and week 8 postsurgery. LVIDd, left ventricular internal dimension at diastole; LVIDs, left ventricular internal dimension at systole; LVPWd, left ventricular posterior wall at diastole; FS, fractional shortening; EF, ejection fraction. ^*∗*^*P* < 0.05, ^*∗∗*^*P* < 0.01, and ^*∗∗∗*^*P* < 0.001 vs. sham group; ^#^*P* < 0.05 and ^##^*P* < 0.01 vs. TAC group. Data are presented as mean ± SEM. *n* = 12 in each group.
