# Supplementary material for: Qindan Capsule Attenuates Myocardial Hypertrophy and Fibrosis in Pressure Overload-Induced Mice Involving mTOR and TGF-β1/Smad Signaling Pathway Inhibition
Source: Evid Based Complement Alternat Med. 2021 Apr 28;2021:5577875. doi: 10.1155/2021/5577875 (PMC8102107; doi:10.1155/2021/5577875)
Supplement: Supplementary Materials — Figure S1: quality evaluation of QC using HPLC: (a) baicalin; (b) 3,4-dihydroxyphenyllactic acid; (c) berberine; (d) rhynchophylline; and (e) stachydrine. Table S1: recipe of Qindan capsule (QC) formulation. Table S2: mouse primers used for real-time RT-PCR. Table S3: echocardiographic parameters in different time points. Cardiac functional parameters measured by transthoracic echocardiography at week 0 (baseline), week 4, and week 8 postsurgery. LVIDd, left ventricular internal dimension at diastole; LVIDs, left ventricular internal dimension at systole; LVPWd, left ventricular posterior wall at diastole; FS, fractional shortening; EF, ejection fraction. ∗P < 0.05, ∗∗P < 0.01, and ∗∗∗P < 0.001 vs. sham group; #P < 0.05 and ##P < 0.01 vs. TAC group. Data are presented as mean ± SEM. n = 12 in each group. [file 5577875.f1.zip › 5577875.f1/Supplement Data Table S1 (1).pdf]

Table S1 Recipe of Qindan-capsule (QC) formulation

| Components | Voucher specimens' number | Part | Weight (g) |
|---|---|---|---|
| *Scutellaria baicalensis* Georgi | SDU0174 | Root | 12 |
| *Salvia miltiorrhiza* Bge | SDU0159 | Root | 30 |
| *Coptis chinensis* Franch | SDU0186 | Rhizome | 9 |
| *Uncaria rhynchophylla (Miq.) Miq. ex Havil* | SDU0177 | Ramulus | 30 |
| *Ligusticum striatum DC* | SDU0132 | Rhizome | 9 |
| *Pheretima aspergillum (*E. Perrier*)* | SDU0213 | Dried body | 12 |
| *Leonurus japonicus Houtt* | SDU0187 | Aerial part | 15 |
| *Scurrula parasitica L* | SDU0166 | Stem and branch | 20 |
