# Supplementary material for: Qindan Capsule Attenuates Myocardial Hypertrophy and Fibrosis in Pressure Overload-Induced Mice Involving mTOR and TGF-β1/Smad Signaling Pathway Inhibition
Source: Evid Based Complement Alternat Med. 2021 Apr 28;2021:5577875. doi: 10.1155/2021/5577875 (PMC8102107; doi:10.1155/2021/5577875)
Supplement: Supplementary Materials — Figure S1: quality evaluation of QC using HPLC: (a) baicalin; (b) 3,4-dihydroxyphenyllactic acid; (c) berberine; (d) rhynchophylline; and (e) stachydrine. Table S1: recipe of Qindan capsule (QC) formulation. Table S2: mouse primers used for real-time RT-PCR. Table S3: echocardiographic parameters in different time points. Cardiac functional parameters measured by transthoracic echocardiography at week 0 (baseline), week 4, and week 8 postsurgery. LVIDd, left ventricular internal dimension at diastole; LVIDs, left ventricular internal dimension at systole; LVPWd, left ventricular posterior wall at diastole; FS, fractional shortening; EF, ejection fraction. ∗P < 0.05, ∗∗P < 0.01, and ∗∗∗P < 0.001 vs. sham group; #P < 0.05 and ##P < 0.01 vs. TAC group. Data are presented as mean ± SEM. n = 12 in each group. [file 5577875.f1.zip › 5577875.f1/Supplement Data Table S3 (1).pdf]

Table S3. Echocardiographic parameters in different time point

	Sham	TAC	TAC+QCL	TAC+QCH	TAC+LST
0 week					
Heart rate (beat/min)	496±13	482±19	513±21	492±14	521±25
LVIDd (mm)	3.11±0.12	3.19±0.13	3.26±0.12	3.24±0.14	3.22±0.13
LVIDs (mm)	1.62±0.12	1.61±0.11	1.61±0.13	1.63±0.13	1.68±0.12
LVPWd (mm)	0.75±0.06	0.78±0.08	0.74±0.08	0.76±0.07	0.77±0.07
FS (%)	48.3±2.2	47.8±2.4	50.9±2.4	49.8±2.5	48.5±2.3
EF (%)	81.3±3.5	83.2±3.9	85.7±4.3	83.6±3.8	80.6±3.5
4 weeks					
Heart rate (beat/min)	517±21	524±24	508±26	499±25	512±21
LVIDd (mm)	3.14±0.13	3.46±0.19	3.37±0.21	3.24±0.16	3.39±0.18
LVIDs (mm)	1.68±0.13	2.12±0.16	1.89±0.17	1.86±0.18	1.92±0.19
LVPWd (mm)	0.78±0.07	1.16±0.12**	0.98±0.09	0.86±0.08#	0.92±0.11
FS (%)	48.3±2.1	38.1±2.3	43.9±2.4	44.3±2.3	43.4±2.5
EF (%)	80.6±3.5	67.7±4.9	71.8±5.6	72.8±5.1	70.3±4.9
8 weeks					
Heart rate (beat/min)	503±27	518±26	531±29	523±27	527±24
LVIDd (mm)	3.21±0.14	3.89±0.16*	3.63±0.15	3.42±0.18	3.74±0.19
LVIDs (mm)	1.69±0.11	2.93±0.17***	2.37±0.18	2.08±0.17#	2.41±0.19
LVPWd (mm)	0.77±0.06	1.29±0.11***	0.93±0.07#	0.85±0.06#	0.91±0.09#
FS (%)	47.3±2.3	24.2±2.6***	34.7±2.5#	38.6±2.8##	35.6±2.9#
EF (%)	80.6±3.9	45.8±4.6***	66.9±4.9#	69.2±5.6#	67.3±5.4#

Cardiac functional parameters measured by transthoracic echocardiography at week 0 (baseline), week 4, and week 8 post-surgery. LVIDd, left ventricular internal dimension at diastole; LVIDs, left ventricular internal dimension at systole; LVPWd, left ventricular posterior wall at diastole; FS,

fractional shortening; EF, ejection fraction. *$P < 0.05$, **$P < 0.01$, ***$P < 0.001$ vs. Sham group; $^{\#}P < 0.05$, $^{\#\#}P < 0.01$ vs. TAC group. Data are mean ± SEM. n = 12 in each group.
